# SARS-CoV-2: characteristics and current advances in research

**DOI:** 10.1186/s12985-020-01369-z

**Published:** 2020-07-29

**Authors:** Yicheng Yang, Zhiqiang Xiao, Kaiyan Ye, Xiaoen He, Bo Sun, Zhiran Qin, Jianghai Yu, Jinxiu Yao, Qinghua Wu, Zhang Bao, Wei Zhao

**Affiliations:** 1 State Key Laboratory of Cardiovascular Disease, Fuwai Hospital, National Center for Cardiovascular Diseases, Chinese Academy of Medical Sciences and Peking Union Medical College, Beijing, 100037 China; 2 Guangdong Provincial Key Laboratory of Tropical Disease Research, School of Public Health, Southern Medical University, Guangzhou, 510515 China; 3 Department of clinical medicine, Zhengzhou university, 100 Science Avenue, Zhengzhou, 450001 China; 4 Second Clinical Medical College, Southern Medical University, Guangzhou, 510515 China; 5 Yang Jiang Hospital, Yangjiang, 510515 Guangdong Province China

**Keywords:** SARS-CoV-2, COVID-19, Cytokine storm, Cardiovascular disease, Myocardial injury, Venous thromboembolism, Treatment

## Abstract

Coronavirus disease 2019 (COVID-19) caused by SARS-CoV-2 infection has spread rapidly across the world and become an international public health emergency. Both SARS-CoV-2 and SARS-CoV belong to subfamily *Coronavirinae* in the family *Coronaviridae* of the order *Nidovirales* and they are classified as the SARS-like species while belong to different cluster. Besides, viral structure, epidemiology characteristics and pathological characteristics are also different. We present a comprehensive survey of the latest coronavirus—SARS-CoV-2—from investigating its origin and evolution alongside SARS-CoV. Meanwhile, pathogenesis, cardiovascular disease in COVID-19 patients, myocardial injury and venous thromboembolism induced by SARS-CoV-2 as well as the treatment methods are summarized in this review.

## Background

The COVID-19 pandemic has resulted in more than 6.6 million confirmed cases worldwide. Previous studies showed that both SARS-CoV-2 and SARS-CoV belong to the subfamily *Coronavirinae* of the *Nidovirales coronaviridae*, and are classified as SARS-like species, but belong to different clusters. To further explore the characteristics of SARS-CoV-2, we compared different aspects of the virus with those of SARS-CoV; the clinical manifestations and treatment methods are also summarized.

## Introduction

Coronaviruses belong to the subfamily *Coronavirinae* in the family *Coronaviridae* of the order *Nidovirales* and can cause respiratory, digestive, and nervous system diseases in humans and many other animals. Coronavirus particles are spherical with a diameter of approximately 80 to 160 mm. The envelope surface is covered with spike (S) protein, and the membrane (M) proteins and envelope (E) proteins are located among the S proteins. The genomic RNA and phosphorylated nucleocapsid (N) protein form a spiral nucleocapsid, which is located within the envelope [1, 2]. The coronavirus genome is comprised of a single-stranded positive-strand RNA ranging from 26 Kb to 32 Kb in length, constituting the longest known genome among RNA viruses [3]. This genome has a 5′ cap structure, a 3′ polyadenylate tail structure, and six open reading frames (ORFs), of which the first (ORF1) near the 5′ terminus encodes 16 non-structural proteins (nsp1–16) involved in viral replication and transcription; other ORFs encode the four major structural proteins (S, M, N, and P) and eight accessory proteins (3a, 3b, p6, 7a, 7b, 8b, 9b, and ORF14), playing an important role in the assembly of viral particles.

According to genetic and antigenic characteristics, coronaviruses can be divided into four genera: α, β, γ, and δ. Among them, α and β coronaviruses only infect mammals, while γ and δ mainly infect birds, although some can also infect mammals [4, 5]. Except for SARS-CoV and Middle East Respiratory Syndrome Coronavirus (MERS-CoV), most coronaviruses do not cause severe diseases in humans. It has been confirmed that the recent outbreak and epidemic of coronavirus disease 2019 (COVID-19) was caused by a new coronavirus that has been named SARS-CoV-2. Different from SARS-CoV and MERS-CoV in genetics and epidemiology, SARS-CoV-2 is a novel β-coronavirus [6, 7]. As of now, three types of highly pathogenic coronaviruses have been confirmed, namely SARS-CoV, MERS-CoV, and SARS-CoV-2 [8].

In our review, we explore the differences in the origin and evolution, amino acid composition and protein structure, epidemiological and pathological characteristics between SARS-CoV-2 and SARS-CoV. In addition, the pathogenesis of SARS-CoV-2 has been summarized. Based on our expertise, comorbidity of cardiovascular diseases (CVD) in COVID-19 patients and SARS-CoV-2-induced myocardial injury and venous thromboembolism (VTE) are fully discussed, and medicines with recent clinical trial outcomes are also introduced.

## Differences between SARS-CoV-2 and SARS-CoV

### Classification

According to the principle of international commission on virus classification, the coronavirus identification mainly depends on the similarity of the amino acid sequences of the seven domains encoded by ORF1ab, including ADRP, nsp5, and nsp12–16. Due to the extremely similar (more than 90%) amino acid sequences in the seven domains, both SARS-CoV-2 and SARS-CoV belong to the subfamily *Coronavirinae* in the family *Coronaviridae* of the order *Nidovirales* and are classified as SARS-like species, although they are classified into different clusters. The former belongs to the bat-like coronavirus cluster and the latter to the SARS cluster. Phylogenetic analysis showed that SARS-CoV-2 has a longer branch length compared to its closest relatives, including bat-SL-CoVZC45 and bat-SL-CoVZXC21; furthermore, it is genetically different from SARS-CoV. SARS-CoV-2 has only 79.5 and 40% homology with SARS-CoV and MERS-CoV, respectively, indicating a large genetic distance. At the same time, the S-protein homology between SARS-CoV and SARS-CoV-2 is also relatively low at 76.5% [9–12].

### Amino acid composition and protein structure

While SARS-CoV-2 is very similar to SARS-CoV in amino acid composition and protein structure, with both having an Orf1ab encoding 16 predicted Nsps as well as the 4 typical coronavirus structural proteins, they also show some differences, mainly in the S, ORF8, ORF3b, and ORF10 proteins, with limited detectable homology between them.

Like SARS-CoV, the entry of SARS-CoV-2 is mediated by the recognition of the receptor binding domain (RBD) in the S protein and the angiotensin converting enzyme 2 (ACE2) receptor on the surface of the host cell, and the activation of S protein is related to TMPRSS2, whose inhibitors can prevent virus invasion [13]. Most of the SARS-CoV-induced polyclonal antibodies can prevent the S-mediated entry of the virus, which further illustrates the similarity between these two coronaviruses. However, according to previous researches, the outer subdomain of the receptor-binding domain in the S protein of SARS-CoV-2 has only 40% amino acid homology with other SARS-associated coronaviruses [3]. A recent research found that Furin protease cleavage site exists at the boundary between the S1 subunit and S2 subunit in the S protein of SARS-CoV-2, and it is processed during the biosynthesis [14]. This is similar to several highly pathogenic avian influenza viruses [15] and pathogenic Newcastle disease virus [16], but distinguishes SARS-CoV-2 from SARS-CoV. The existence of the cleavage site of the Furin protease enhances the tissue and cell tropism and transmissibility of SARS-CoV-2, and alters its pathogenicity. Wrapp et al. [17] obtained the trimeric structure of the S protein by 3D reconstruction technology based on the genomic sequence of SARS-CoV-2, and found that it is structurally different from that of SARS-CoV. In addition, the affinity of S protein of SARS-CoV-2 to ACE2 increased by 10–20 times compared with that of SARS-CoV. Blocking the process of viral entry is an important way to prevent and control viral infections; identifying and understanding the protein molecules on the surface of the new coronavirus, related receptors of target cell, as well as their interaction mechanisms can provide a basis for effectively preventing viruses from invading host cells. RBD is recognized primarily via polar residues by the extracellular peptidase domain of ACE2. Yan et al. [18] analyzed the electron microscope structure of the complex of S protein and ACE2, and found that in the procedure of the virus-target cell binding, the loop region on RBD crossed the α1 helix of ACE2, and the loop regions of β3, β4 and α2 helix are also involved in the combination of RBD and ACE2. Superimposing of the structures of SARS-COV-RBD and SARS-COV-2-RBD suggests a very high degree of similarity between the two, but there are still differences. The R426, Y484, T487, V404, and L472 residues in SARS-COV-RBD were replaced by N439, Q498, N501, K417, and F486 in the SARS-COV-2-RBD respectively. The replacement of L472 by F486 will enhance the van der Waals effect, and that of R426 by N439 will eliminate the salt bridge effect of D329 in ACE2, which, however, would be strengthened when V404 is replaced by K417. The existence of these mutations may be a significant reason SARS-COV-specific RBD antibody drugs fail to work on SARS-COV-2.

The motif VLVVL (amino acids 75–79) was reported in SARS-CoV ORF8b, which can trigger the intracellular stress pathway and activate the NLRP3 endosome, while no functional domains containing this motif has been found in SARS-CoV-2. ORF8 is related to the evolution of SARS-associated coronaviruses, and plays a significant role in virus replication, transmission, and adaptation to its hosts. In SARS-CoV-2, ORF8 consists of 121 amino acids, while it exists as ORF8a (39 amino acids) and ORF8b (84 amino acids) in SARS-CoV. The ORF3b protein contains 154 amino acids in SARS-CoV, but only 67 amino acids in SARS-CoV-2. In addition, the ORF3b protein of SARS-CoV-2 contains four new helical structures, and shows no homology to that of SARS-CoV. Although ORF3b protein is not necessary for virus replication, it may be related to its pathogenicity and its importance in SARS-CoV-2 requires further study [19].

Recently, a study carried out by multiple teams in the United States, France, and the UK cloned, tagged and expressed 26 of the 29 SARS-CoV-2 proteins in human cells, and suggested that ORF10 of SARS-CoV-2 shows limited homology with that of SARS-CoV. The study found that ORF10 of SARS-CoV-2 is small in size (38 amino acids), but contains an alpha helical region, which can be linked to a Cullin 2 (CUL2) RING E3 ligase complex, especially the CUL2ZYG11B complex, and hijack it for ubiquitination and restriction factor degradation. Alternatively, ZYG11B may bind to the N-terminal glycine in Orf10 to target it for degradation, which is closely related to virus replication [20].

**Fig. 1:**
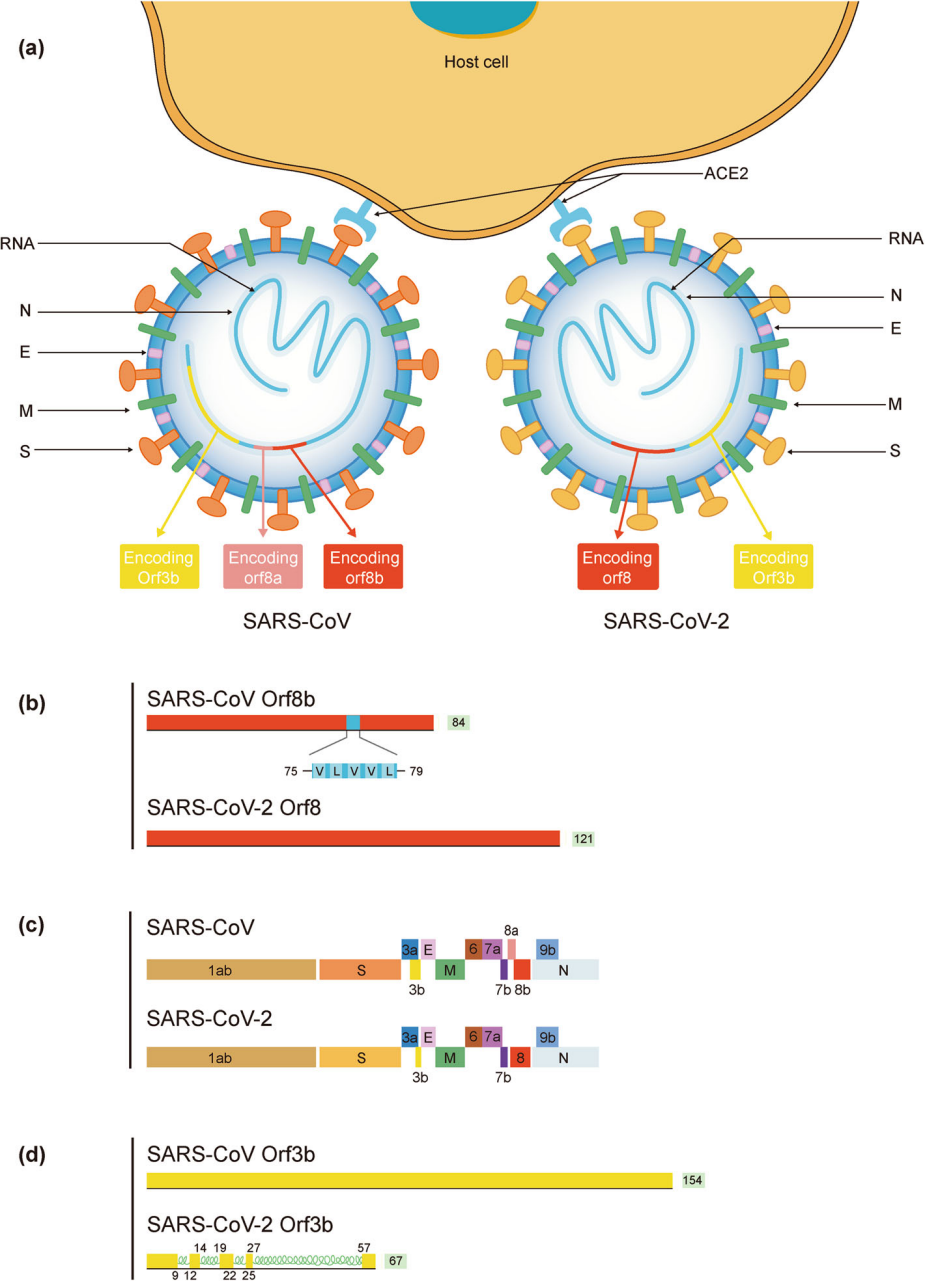
Distinctions of amino acid composition and protein structure. Differences between SARS-CoV and SARS-CoV-2 are mainly in S protein, ORF8 protein and ORF3b protien. **a** The external subdomain of the receptor binding domain of the spike protein in SARS-CoV-2 shares only 40% amino acid identity with other SARS-related coronaviruses; **b** ORF8 in SARS-CoV-2 does not contain a known functional domain or motif while in SARS-CoV ORF8b the presence of the aggregation motif VLVVL has been found; **c** The ORF8a protein is absent in SARS-CoV-2; There are 121 amino acids that encode the 8b protein in SARS-CoV-2, while only 84 are involved in SARS-CoV. **d** ORF3b of SARS-CoV-2 has a novel protein with four helices and 67 amino acids that encode the 3b protein in SARS-CoV-2 while 154 amino acids are involved in SARS-CoV

Currently, it is believed that the SARS-CoV-2 genome is more stable than SARS-CoV, but it is still necessary to strengthen the monitoring of viral genome mutations as the epidemic progresses. Some large-scale viral genome studies suggest that 149 mutation sites have appeared in SARS-CoV-2. Due to the sequence difference in site 28,144 in the viral RNA genome, SARS-CoV-2 is divided into two subtypes: L and S. The L type spreads more widely, and has more mutations and a stronger ability to spread. Compared with other coronavirus, the gene sequence of S protein in SARS-CoV-2 changes greatly, suggesting that this segment may show a higher mutation rate [21]. Su et al. used a second-generation sequencing platform to analyze the nasal swab samples of patients diagnosed with COVID-19 and found that the 3′-end of the SARS-CoV-2 genome has a fragment with 382 nt missing, resulting in the destruction of the function of the ORF8 region, which may relate to how SARS-CoV-2 adapts to human survival [22]. Distinctions in the structure between SARS-CoV and SARS-CoV-2 are shown in Fig. 1.

### Epidemiological characteristics

Studies indicate that SARS-CoV has an incubation period of 2 to 10 days and a median incubation period of 4 to 7 days, while the incubation period of SARS-CoV-2 is mostly within 14 days, and the median is 3–4 days.

#### Sources of infection

The SARS epidemic in 2003 first occurred in Guangdong Province. Sources of SARS-CoV infection include infected animals and humans. At present, it is generally believed that the virus originates from bats, and civet is a possible intermediate host, and humans are the final hosts [23].

At the end of 2019, the first outbreak of pneumonia caused by SARS-CoV-2 occurred in Wuhan, Hubei [24]. Besides infected animals and COVID-19 patients, asymptomatic infectors are the most important source of infection for SARS-CoV-2 [25]. Studies have demonstrated that SARS-CoV-2 is of bat origin [12, 26], with pangolin or civet as one of the possible intermediate hosts, and humans are the ultimate hosts [27]. It is worth noting that a recent study isolated one coronavirus from a Malayan pangolin showing 100, 98.6, 97.8, and 90.7% amino acid identity with SARS-CoV-2 in the E, M, N and S genes, respectively, and the receptor-binding domain within the S protein of the Pangolin-CoV is virtually identical to that of SARS-CoV-2, with only one noncritical amino acid difference. This suggests that SARS-CoV-2 might have originated from the recombination of a Pangolin-CoV-like virus with a Bat-CoV-RaTG13-like virus [28]. However, more research is required to confirm this.

#### Routes of transmission

SARS-CoV is transmitted through close-up droplets and contact, while SARS-CoV-2 has a wider range of transmission routes. In addition to short-distance droplet transmission and contact transmission, SARS-CoV-2 can also be transmitted through aerosols in the enclosed space and urine, and mother-to-child transmission may also exist [29–31]. The Chinese Center for Disease Control and Prevention isolated the SARS-CoV-2 strain from a feces sample of a confirmed patient in Heilongjiang Province, indicating that SARS-CoV-2 can survive in the stool; it has been demonstrated that after intragastric administration of SARS-CoV-2, transgenic mice that express human ACE2 can get the infection and show related pathological changes [32]. This suggests that fecal-oral transmission may also be one of its transmission modes [29, 33].

#### Susceptible population

The population is generally susceptible to SARS-CoV, mostly young adults; and people are also generally susceptible to SARS-CoV-2. Epidemiological analysis shows that 77.8% of patients with COVID-19 are between 30 and 69 years of age, with the highest proportion in the 50 to 60 years age group, while the infection rate of children is relatively low [8, 34].

It is generally believed that SARS-CoV-2 has a stronger propagation capability than SARS-CoV. A previous study showeed basic reproduction number (R_0_) was 2.9 [35, 36]. Based on the epidemiological data of 425 patients, another study showed the basic reproduction number R0 of this new coronary pneumonia to be 2.20 [37, 38]. Moreover, there is a study that predicts the R0 value of SARS-CoV-2 to be 3.28 [39]. Yang et al. [40] predicted the R_0_ to be 3.77—higher than SARS-CoV—but because of the uncertainty, the accuracy of the estimate is limited. The latest research shows that the R_0_ of SARS-CoV-2 is about 2.68, which is roughly similar to the R0 reported by World Health Organization (WHO) and the Chinese Center for Disease Control and Prevention [41, 42]. SARS-CoV-2 is highly contagious and up to June 17, 2020, SARS-CoV-2 infection had occurred in as many as 216 countries and the cumulative number of COVID-19 patients globally had reached 8,061,550, according to the data from WHO.

SARS-CoV-2 spreads easily but it is less lethal. The mortality rate of SARS-CoV-2 was lower than that of SARS-CoV. Studies have shown that approximately 23 to 32% of patients with SARS will develop severe disease and are prone to death [43]. A report by WHO shows that 774 of 8098 SARS patients died, with a case fatality rate of 9.6%. In elderly patients, case fatality rate was up to 50% [36]. SARS-CoV-2 has a wider range of transmission than SARS-CoV or MERS-CoV, and infects a larger number of patients, but the ratio of critically ill COVID-19 patients is relatively lower. Epidemiological characteristics of more than 70,000 cases described that 80.9% COVID-19 patients presented mild/moderate illness. Meanwhile, the crude death rate of COVID-19 was 2.3% and the death rate was 0.015/10 person-days [34], much lower than the mortality rates of SARS [44]. Severe illness and death are more common in older patients with underlying conditions. Meanwhile, SARS-CoV-2 not only affects the lungs, but also the heart and kidneys, causing multiple organ failure. Consequently, therapy for severe COVID patients is more difficult than that for SARS.

#### Origin and evolution

During the widespread epidemic of SARS-CoV-2 in the world, analysis of the SARS-CoV-2 genomic system evolution network revealed three variants, which the researchers tentatively named A, B, and C. Among them, A is an ancestor type; B is derived from A through two mutations of the synonymous mutation T8782C and the non-synonymous mutation C28144T, which is derived from A. The difference between type C and its parent type B is the non-synonymous mutation G26144T, and this mutation converts glycine to valine. Notably, the different types have different geographical distributions in the world. A and C mainly exist in Europe and the United States, while B mainly exists in East Asia, suggesting that Wuhan, the first outbreak spot of type B SARS-CoV-2 may not be the origin of SARS-CoV-2. This provides a new idea for the origin and evolution of SARS-CoV-2 [45].

### Pathological characteristics

Autopsy results in SARS patients suggest that SARS-CoV infection causes severe pulmonary edema, pulmonary congestion, hilar lymphadenopathy, and spleen shrinkage in general [46, 47]. Histological features of patients with SARS include bronchial epithelial exfoliation, loss of cilia and squamous metaplasia, diffuse alveolar damage, formation of hyaline membranes, and severe fibrosis of the lung tissue. SARS-CoV can be detected in lymphocytes, monocytes, lymphoid tissues, and respiratory tract as well as in intestinal mucosa, renal tubular epithelial cells, and neurons [48, 49].

Certain pathological characteristics of COVID-19 patients have been identified. Pulmonary pathological results attained by Tian et al. [50] suggested that the early pathological changes induced by SARS-CoV-2 pneumonia include pathological interstitial pneumonia and prominent pulmonary edema, with protein exudation and minor inflammatory cell infiltration. Xu et al. [51] performed a case dissection on a patient and the results showed that bilateral diffuse alveolar damage with cellular fibromyxoid exudates and hyaline membrane formation corresponded to acute respiratory distress symptoms (ARDS); moreover, the overall pathological characteristics of the lung were similar in SARS and MERS. Inflammatory infiltration of lymphocyte-dominated mononuclear cells and other viral cytopathic-like changes were seen in the lung, but no intranuclear or intracytoplasmic viral inclusions were found. Minor inflammatory infiltration of mononuclear cells was present in the myocardial interstitium and the existence of viral myocarditis could not be ruled out, but no obvious damage to the myocardium was found. Based on the pathology report of one particular patient, the impact of SARS-CoV-2 on the cardiovascular system cannot be determined; hence, additional sample research and analysis are necessary. Another autopsy of a COVID-19 patient in China found that mucus exudation was more obvious than that in SARS patients and lung damage involving diffuse alveolar damage and pulmonary hyaline membrane formation was serious. Histopathologic changes seen on postmortem transthoracic needle biopsies from a COVID-19 patient with hypertension and diabetes showed diffuse alveolar damage. Virus was more highly detected in alveolar epithelial cells, while viral protein expression was low in blood vessels or in the interstitial areas [52]. A recent pathological study of African American patients found that in addition to diffuse alveolar injury, inflammatory cell infiltration, and hyaline membrane formation, COVID-19 patients also have thrombi in peripheral small vessels with obvious bleeding in the lungs, while no obvious thrombus was found in other organs, including kidney, spleen, pancreas, and liver. At the same time, it was reported that there are a large number of CD61^+^ megakaryocytes in the alveolar capillaries, and a large number of platelets are actively produced. The large aggregation of platelets and fibrin deposition may jointly promote the production of thrombi within peripheral small vessels in lungs [53]. However, evidence of damage to other organs and/or systems requires more substantial autopsy results.

The differences between SARS-CoV and SARS-CoV-2 are shown in Table 1.

**Table 1:** Characteristics of SARS-CAoV and SARS-CoV-2

Differences		SARS-CoV	SARS-CoV-2
**Virological characteristics**	**Classification**	the SARS cluster	bat-like coronavirus cluster
	**Amino acid composition of ORF8b**	aggregation motif VLVVL in SARS-CoV ORF8b	no functional domain or motif in SARS-CoV-2 ORF8b
	**Number of amino acids encoding 8b protein**	84	121
	**Number of amino acids encoding 3b protein**	154	67
	**ORF3b protein**	/	having four helices
	**8a protein**	existing	nonexistent
**Epidemiological characteristics**	**sources of infection**	wild animals, patients	wild animals, patients and asymptomatic infector
	**intermediate host**	civet	Pangolin?
	**routes of transmission**	respiratory tract transmission, contact transmission	respiratory tract transmission, contact transmission, may be including digestive tract transmission?, urine? and mother-to-child transmission?
	**susceptible populations**	susceptibility to the entire population, mainly in adults	susceptibility to the entire population
	**Infectiousness(R**_**0**_**)**	2–3	1.2–3.58 [42, 136–139]
	**crude death rate**	11%	1.4–7.0% [140–143]
**Clinical characteristics**	**respiratory symptoms**	High fever, cough are common	low fever, and temperature does not rise in some cases, cough
	**Gastrointestinal symptoms**	Common (diarrhea is rare)	uncommon; can be the initial symptoms
	**cardiovascular symptoms**	/	palpitations and chest pain can be the initial symptoms
	**concominant diseases**	including cardio-cerebrovascular diseases, hypertension, diabetes	higher frequency including cardio-cerebrovascular diseases, hypertension, diabetes
	**imaging features**	pulmonary consolidation and exudation	ground glass opacity, pulmonary consolidation and exudation
**pathological characteristics**	**lung**	pulmonary edema accompanied by exudation of fibrin; Pulmonary fibrosis is a common characteristic.	pulmonary edema accompanied by exudation of fibrin; mainly mucus exudation; thrombi within peripheral small vessels in lungs.

We compare the different aspects including characteristics of virology, epidemiology, clinical manifestations and pathology between SARS-CoV-2 and SARS-CoV in the table in order to provide a further understanding of the virus. ORF: open reading frame; R_0_: basic reproduction number

## Pathogenic mechanisms of SARS-CoV-2

As in SARS-CoV, the S protein of SARS-CoV-2 aids in cell invasion by binding to ACE2 receptors on the host cell surface, causing a series of lung injury responses [54].

### SARS-CoV-2-induced direct damage

When SARS-CoV-2 invades the human body, the RBD on the S1 subunit of the S protein binds to ACE2 expressed on the host cell surface. Subsequently the conformation of the S protein undergoes a significant structural rearrangement, resulting in shedding of the S1 subunit and transition of the S2 subunit to a highly stable post-fusion conformation, which in turn mediates the fusion of the virus with the host cell membrane and cell entry [2]. After entering the cell, SARS-CoV-2 multiplies and eventually lyses the host cell, causing extensive alveolar damage and ARDS in infected patients.

### Down-regulation of ACE2

In addition to mediating the entry of SARS-CoV and SARS-CoV-2 into host cells, ACE2 is also an important mediator of inflammation in the human body. It is mainly expressed in the small intestine, testis, adipose tissue, kidney, heart and thyroid, and lung tissue in the human body, and is also expressed in relatively low amounts in the colon, liver, bladder and adrenal glands, blood, spleen, bone marrow, brain, blood vessels, and muscles [55]. ACE2 is an enzyme that converts angiotensin (Ang) I to Ang 1–9, Ang II to Ang 1–7, and the latter can interact with MAS receptors, thereby inhibiting the harmful vasodilation and pro-fibrosis mediated by the AT1 receptor and mediating a variety of beneficial negative feedback regulation [56]. A lack of ACE2 will increase the levels of the two Ang peptides, thereby activating the Ang AT 1 and AT 2 receptors expressed on the surface of alveolar epithelium, vascular endothelium, intestinal epithelium, and kidney cells. During the fusion of the viral envelope with the host cell membrane and cell entry, ACE2 is internalized accordingly due to its binding to the virus, thereby down regulating ACE2 on the cell surface [56]. The dysregulation of the ACE2-Ang II-AT1 receptor axis and the ACE2-Ang1–7-Mas receptor axis is an important cause of endothelial cell damage, inflammation, and thrombosis [55].

### Immune dysfunction

The disturbance of the immune system is also one of the factors that contributes to tissue and cell damage in patients with COVID-19. In both COVID-19 patients and animal models of SARS-CoV-2 infection, significant inflammatory cell infiltration, increased inflammatory mediators, thickened alveolar septa, and significant vascular system damage have been observed [32]. At present, pathological reports indicate that severe immune injury is an important pathogenic mechanism of SARS-CoV-2.

#### Cytokine storm

A large number of studies have shown that the progression of severe COVID-19 patients is closely related to the massive production and activation of cytokines and inflammatory mediators. The inflammatory response is strong during SARS-CoV-2 infection, and the uncontrolled inflammation of the lungs caused by it may be the main cause of death in some cases. Intensive care unit (ICU) patients have higher levels of interleukin (IL)-1β, IL-1Ra, IL-7, IL-8, IL-9, IL-10, basic FGF, GCSF, GM-CSF, IFN-γ, CXCL10, CCL2, CCL3, CCL4, PDGF, TNF-α, and VEGF in the plasma than healthy controls, and higher levels of IL-2, IL-7, IL-10, GCSF, CXCL10, CCL2, CCL3, and TNF than non-ICU patients [57]. In addition, neutrophils, elevated D-dimers, and blood urea nitrogen were found in deceased patients infected with COVID-19, suggesting that death may be the result of cytokine storms, inflammatory responses, and acute kidney injury [58]. Nlrp3γ inflammasome, as a powerful pro-inflammatory system in the body, is also an important cause of cytokine storm. Nlrp3γ is expressed in many cells, including immune, endothelial, hematopoietic, lung epithelial, kidney, and heart cells. High levels of Ang II may over-activate Nlrp3γ in these cells and trigger an immune response through intracellular caspase-1, thereby releasing a large number of inflammatory factors, such as IL-1β and IL-18, and creating gasdermin D pore channels in cell membranes to mediate the release of several biologically active danger-associated molecular pattern molecules, finally mediating cell apoptosis and lysis [56].

#### Activation of complement system

The complement system is also involved in immune injury in COVID-19 patients. In the peripheral blood mononuclear cells of COVID-19 patients, the genes related to complement activation are enriched, and the serum complement levels in patients with severe COVID-19 are higher than those in mild cases and healthy controls, indicating that complement-mediated immune injury may be one of the causes of cell damage and aggravation of the disease in patients with COVID-19 [59, 60]. Mannose-binding lectin (MBL), a pattern recognition protein present in serum can be combined with MBL-associated serine protease 2 (MASP-2) to initiate the complement-activated lectin pathway by binding to sugar molecules on the surface of pathogens. The SARS-CoV-2 N protein can interact with MASP-2, inducing MASP-2 to automatically activate and cleave complement protein C4 [59]. Massive deposition of MBL, MASP-2, and C3 and C4 lysates (C4a, C4d) in lung tissue and the membrane attack complex formed with C5b-9 can cause damage and lysis of alveolar cells.

#### Lymphocyte dysfunction

Lymphopenia, a common feature in patients with COVID-19, was identified in a patient by flow cytometry while lymphocytes were found to be over-activated. This potentially constitutes a key factor related to disease severity and mortality. The number of CD4^+^ and CD8^+^ T cells in the peripheral blood of patients was greatly reduced, while a higher number of double positive HLA-DR and CD38 suggested the activation of T cells. In addition, the number of CCR4^+^ CCR6^+^ Th17 cells with a high pro-inflammatory effect was increased and CD8^+^ T cells had a high concentration of cytotoxic granules including perforin and granulysin. Over-activation of T cells characterized by an increase in Th17 and high cytotoxicity of CD8^+^ T cells could partially explain the severe immune damage in SARS-CoV-2-infected patients. Viral infection rarely caused a Th17 response, but over-activation of Th17/CD8 was detected in patients with COVID-19 requiring medical attention.

However, the latest research indicates that patients with severe COVID-19 also have impaired cytotoxic lymphocyte killing function [61]. All subtypes of lymphocytes in patients with COVID-19 are reduced, including T cells, B cells, and NK cells. In quantitative analysis of CD4^+^ and CD8^+^ T cells at different stages of maturity [61], it was found that compared with healthy subjects, the frequency of TEMRA (CD45RA^+^ CCR7^−^) and senescent CD8^+^ T cells (CD57 ^+^) in COVID-19 patients was significantly higher. However, Tem (CD45RA7^−^CCR77^−^) and HLA-DR^+^ CD8^+^ T cells did not show related changes compared with healthy subjects, exhibiting a skewing of CD8^+^ T cells towards a terminally differentiated/senescent phenotype; similar findings were also observed for CD4^+^ T cells. Research on NK cells showed that, in addition to a reduced number of the cells in patients with COVID-19, their ability to produce IFN-α, perforin, and granzymes was also reduced, leading to an impaired virus clearance function. IL-6 may play a major role in the process of the dysfunction of NK cell [61, 62]. In patients with severe COVID-19, the decrease in NK cells and their dysfunction are significantly inversely proportional to the level of IL-6 in the serum, while anti-IL-6 receptor monoclonal antibody tocilizumab treatment is able to reverse this process, suggesting that high levels of IL-6 exposure can down-regulate the expression of perforin and granzyme in NK cell. In conclusion, the senescence of CD4^+^ and CD8^+^ T cells as well as the impaired function of NK cells can lead to the evasion of SARS-CoV-2 from the immune attack and clearance in patients with severe COVID-19.

## Clinical manifestations

### Basic clinical characteristics of COVID-19

SARS-CoV-2 infection causes systemic and respiratory symptoms such as fever, muscle soreness, cough, and dyspnea. Guan et al. [30] collected data of 1099 confirmed COVID-19 patients from 552 hospitals in 30 provinces, autonomous regions, and municipalities in China and demonstrated that cough (67.8%) is the most common symptom among patients, while only 43.8% of patients were diagnosed with fever. ARDS, respiratory failure, multiple organ dysfunction syndrome, as well as septic shock, metabolic acidosis, and coagulation dysfunction were found to manifest in severe cases. Meanwhile, nausea, vomiting, diarrhea, and other gastrointestinal symptoms as well as chest pain, heart palpitations, and other cardiovascular symptoms can also be the first symptoms in patients with COVID-19. Laboratory tests show normal or decreased peripheral blood leukocytes, reduced lymphocyte counts, and abnormalities in liver enzymes, myocardial enzymes, and C-reactive protein. In severe cases, increases in D-dimer and inflammatory factors are detected. Computerized tomography showed that ground-glass opacity is the most common radiologic characteristic and “paving stone sign” may appear in the advanced stage [63, 64]. At present, the diagnosis is primarily based on the pathogenic examination of nucleic acid detection. However, nucleic acid detection is subject to factors such as material selection, which may cause a certain false negative rate. Therefore, patients presenting epidemiological characteristics, clinical manifestations, and typical imaging characteristics with negative nucleic acid detection are classified as clinically confirmed cases that must be treated in isolation in the clinic.

### COVID-19 and CVD

Many studies report that patients with COVID-19 often have comorbidities—commonly CVD. Based on the published data in China, the prevalence of CVD in COVID-19 patients varied from 1% [65] to 39% [66]. CDC COVID-19 Response Team analyzed the data from 50 U.S. states, four U.S. territories, and affiliated islands and showed that 9.0% of patients were suffering from CVD [67]. Buckner et al. [68], however, demonstrated that the ratio reached to 38% in Washington State. Mehra et al. [69] enrolled 8910 patients with COVID-19 from 169 hospitals in Asia, Europe, and North America and showed that 10.2% of the patients had coronary artery disease. Meta-analysis confirmed considerable prevalence of CVD among COVID-19 patients. Li et al. [70] reported that the prevalence of cardia-cerebrovascular diseases in COVID-19 patients was 16.4% and another study [71] showed 11.9% of patients with COVID-19 also had CVD. Various proportions of COVID-19 patients with CVD have been singled out due to selection bias and different data samples. It is also worth noting that various definitions of CVD were used in the different studies. For example, some studies recognized coronary heart disease and heart failure as CVD, while some also included cerebrovascular disease and hypertension. Therefore, these results should be cautiously interpreted [72]. Broader data analysis with uniform definition for CVD remains necessary to determine the proportion of COVID-19 patients with CVD.

CVD is regarded as a risk factor of COVID-19 progression and is associated with higher risk of mortality of patients with COVID-19. A previous cross-sectional study reported that COVID-19 patients with CVD and hypertension were more likely to be transferred to the ICU [58]. Furthermore, the co-incidence of COVID-19 with coronary heart disease (5.8% vs. 1.8%) was higher in patients with severe COVID-19 than in non-severe patients [30]. Studies [73, 74] found that CVD was associated with disease severity(OR = 3.14; 95% CI 2.32–4.24; OR = 2.74; 95% CI 1.50–5.00) and also the higher prevalence of CVD in critical/mortal COVID-19 patients compared to the non-critical group was shown(OR = 4.78, 95% CI = 2.71–8.42) in another latest study [75]. The Chinese Center for Disease Control and Prevention announced that the crude mortality of COVID-19 was approximately 0.9%, while in patients with CVD, it rose to 10.5%. Zhang et al. [74] enrolled 541 patients with COVID-19 and showed the mortality of patients with CVD reached to 22.2%. Presence of CVD was associated with higher mortality (OR = 4.85, 95% CI 3.07–7.70). These studies suggest that more intensive medical care should be provided to patients with COVID-19 having CVD to prevent disease progression and poor prognosis [76].

### COVID-19 and myocardial injury

**Fig. 2:**
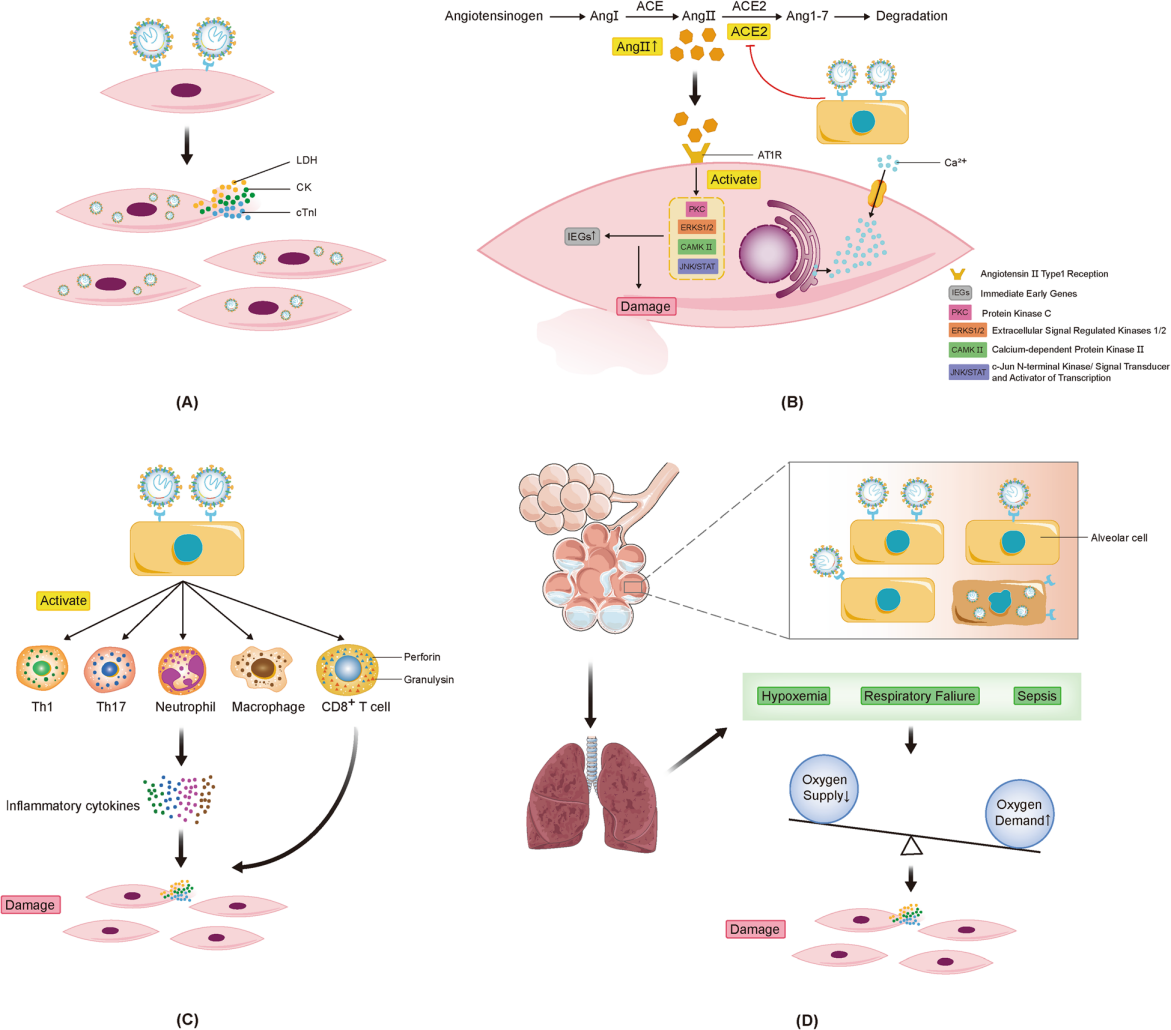
Potential mechanisms of myocardial injury induced by SARS-CoV-2. **a** SARS-CoV-2 damages cardiomyocytes directly; **b** SARS-CoV-2 infection reduces ACE2 thus AngII is up-regulated. Kinases in cardiomyocytes are activated to induce an inflammation effect causing myocardial injury; **c** Inflammatory cytokines release; **d** Oxygen supply-demand imbalance

Myocardial injury is one of the most common complications in patients with COVID-19, especially those in severe condition, with rates reported variously and often indicating a poor prognosis. The prevalence of patients with myocardial injury complication varies from 7.2% [58] to 27.8% [77]. In our upcoming meta-analysis, 7 studies were included and the analysis indicated that the pooled prevalence of myocardial injury complication in COVID-19 patients is 17.0%. In addition, myocardial injury is more commonly seen in severe cases. Huang et al. [57] and Wang et al. [58] demonstrated that the incidence of myocardial injury was 30.7 and 22.2%, respectively. Li et al. [78] showed that the ratio was 34.9% in severe patients. Moreover, it has been proved that myocardial injury is associated with higher risk of in-hospital mortality. The mortality was 51.2% in myocardial injury group, while that in patients without myocardial injury was 4.5% (P < 0.001) [79]. Similar results were also demonstrated in other studies [80–82]. Although the specific mechanisms by which SARS-CoV-2 causes myocardial injury remain unclear, they may be related to the following:

i. **Direct damage** Due to the wide expression of ACE2 receptors in cardiomyocytes, a large number of SARS-CoV-2 may directly invade cardiomyocytes through binding to the receptor, which may cause the cardiac damage. Besides, replication and reproduction of SARS-CoV-2 rely on substrates in cardiomyocytes, which may lead to abnormal metabolism of cardiomyocytes and consequent damage.
ii. **Down-regulation of ACE2** Levels of Ang II, an inflammatory factor regulatory protein, are elevated by SARS-CoV-2 infection, leading to the production of reactive oxygen species and oxidative stress injury of myocardial cells [83, 84]. After Ang II recognizes the AT 1 receptor, several kinases, including extracellular regulated protein kinases 1/2, c-Jun N-terminal kinase/signal transducer and activator of transcription, calcium kinase II and protein kinase C, are also activated. In addition, the down-regulation of ACE2 caused by SARS-CoV-2 activates the ADAM-17/TACE pathway, which leads to increased release of TNF-α and subsequent myocardial inflammatory damage [85, 86]. However, changes in the content of Ang II and ACE2, the initiating factors, and the specific molecular mechanisms that damage the body, require further study.
iii. **Immune damage and cytokine storm** Similar to SARS-CoV and MERS-CoV, SARS-CoV-2 induces the release of a large number of cytokines, causing a cytokine storm that damages myocardial cells [87]. TNF-α, produced by activated macrophages, may be a main chemokine in patients with COVID-19, causing the release of a series of pro-inflammatory factors that also plays an essential role in myocardial damage [88]. The specific molecular mechanisms involving immune damage and cytokine storms with myocardial damage must be studied further.
iv. **Oxygen supply-demand imbalance.** Pulmonary pathology suggests that SARS-CoV-2 infection is mainly due to exudative changes, leading to hypoxemia or respiratory failure. In addition, patients with COVID-19 have systemic symptoms such as fever, leading to increased oxygen demand, which further exacerbates the imbalance between oxygen supply and demand. Mitochondrial damage and oxidative stress induced by the imbalance between oxygen supply and demand are important pathophysiological mechanisms of cardiac damage caused by viral infection [89, 90]. It is speculated that myocardial damage induced by SARS-CoV-2 may also be related to oxidative stress. Mitochondrial structure and function are dysfunctional under hypoxic conditions, the production of antioxidant substances is reduced, and the level of reactive oxygen species is increased, which induces myocardial damage. In addition, endoplasmic reticulum stress induced by hypoxia promotes increase of pro-apoptotic factors and expression of apoptosis gene through activation of PERK-ATF4-CHOP (protein kinase R-like endoplasmic reticulum kinase-transcription activator 4-C/EBP homologous protein) pathway, thereby inducing cardiomyocyte apoptosis and myocardial injury [91]. The different mechanisms of myocardial injury induced by SARS-CoV-2 are shown in Fig. 2.

### COVID-19 and VTE

SARS-CoV-2-induced hypercoagulability and VTE have received great attention recently [92, 93]. Middeldorp et al. [94] showed that 19.6% pf patients with COVID-19 show VTE complication. Llitjos et al. [95], Klok et al. [96], and Cui SP et al. [97] demonstrated that the prevalence of VTE was up to 69.2, 27, and 24.7%, respectively, in ICU-COVID-19 patients. It has been shown that the level of D-dimer is higher in COVID-19 patients [98]. Compared with the non-severe patients of COVID-19, a higher proportion of elevated D-dimer was observed among severe cases [99, 100] and higher level of D-dimer is one of the risk factors for disease progression [101–103]. Besides, higher concentrations of D-dimer (aHR = 1.10 [1.01–1.19] per decile increase) were independently associated with in-hospital mortality [104]. A retrospective cohort study also demonstrated that D-dimer > 1 μg/mL on admission was associated with higher risk of death (OR = 18.4, 95% CI: 2.6–128.6, p = 0.003) [105]. SARS-CoV-2 induces endothelial injury and cytokine storm may explain the appearance of VTE and elevated D-dimer in COVID-19 patients [106, 107] while the exact molecular mechanisms still need to be elucidated. In clinics, VTE and dynamic changes in D-dimer levels should be considered to prevent the clinical deterioration of patients with COVID-19.

## COVID-19 therapy

Treatment of COVID-19 patients is fully discussed nowadays and with worldwide researchers’ efforts, effective therapy strategies are shared to improve the prognosis of patients with COVID-19. Therapies including medicine, specific immunotherapy and cell therapy are expected to play an effective role in treating COVID-19 patients. Here, based on recent research and/or Chinese experience, we comprehensively introduce some effective treatments of patients with COVID-19.

### Medicine therapy

#### Traditional Chinese medicine (TCM)

TCM shows encouraging results in improving symptoms and decreasing the deterioration, mortality, and recurrence rates of COVID-19. In China, 91.5% of patients with COVID-19 have used TCM and efficiency exceeded 90%. Chinese scholars have proposed that TCM can modulate the dysfunction of ACE2 caused by viral infection in multiple pathways. Moreover, it can inhibit ribosomal proteins to obstruct viral replication, conferring a protective effect in humans. Additionally, TCM inhibits the excessive production of activated cytokines and eliminates the inflammatory response by regulating Th17 and cytokine-related pathways, which may provide protective effects in COVID-19 patients. A recent study showed that 8 core herbal combinations and 10 new formulae were regarded as potentially useful candidates for COVID-19 treatment [108]. Lianhuaqingwen capsule, which is a repurposed marketed Chinese herb product has been confirmed for influenza treatment. A prospective multicenter open-label randomized controlled trial [109] proved that it could also be considered to ameliorate clinical symptoms of COVID-19 after 14 days of use. However, further assessment through double blind and longer follow-up duration trials is necessary.

#### Chloroquine and Hydroxychloroquine

Chloroquine and hydroxychloroquine are used for treating malaria and whether they can be a potential drug for COVID-19 treatment is currently controversial. A previous study showed that the use of chloroquine in 100 COVID-19 patients was potentially able to inhibit the virus and it was suggested to be used in clinical treatment in Chinese guideline. Hydroxychloroquine, as an analog of chloroquine, was shown to have a stronger inhibitory effect on SARS-CoV-2 than chloroquine in vitro experiments with a higher safety. In addition, low-dose hydroxychloroquine may also play an immunoregulatory role in severely infected patients who cannot use glucocorticoids and immunosuppressants, and relieve the cytokine storm [110]. Conversely, a recent study demonstrated that there was no evidence for the efficacy of chloroquine or hydroxychloroquine against COVID-19. Furthermore, it increased the risk of serious cardiac complications and mortality of patients. However, because of the high dose of chloroquine or hydroxychloroquine used in this study as well as suspicion on data sources and data consistency, these results cannot be used to reach consensus. Notably, the article was retracted by Lancet [111]. Thus, the clinical value of repurposing these drugs for COVID-19 therapy still requires further investigation and high-quality research.

#### Remdesivir

Remdesivir, an adenosine nucleotide analogue prodrug having broad-spectrum antiviral activity, is expected to become a potent drug for COVID-19 [112]. However, two recent randomized controlled trials showed contradictory results. A Chinese study [113] enrolled 237 severe COVID-19 patients and demonstrated that compared with the placebo group, no improvement in mortality was found after taking remdesivir for 28 days (13.9% versus 12.8%). This study failed to complete full enrollment due to the end of the disease outbreak and 2:1 randomization in trial, leading to lower inspection efficiency, both of which may decrease credibility of the conclusion. A larger American study [114], which included 1063 patients with COVID-19, demonstrated that remdesivir was effective for treatment. It showed that median recovery time in remdesivir group was 11 days compared to 15 days in the placebo group (P < 0.001), and 14-day mortality was 7.1 and 11.9% in remdesivir group and placebo group, respectively. Researchers are optimistic regarding the use of remdesivir for COVID-19 treatment, although more clinical trials are required to provide strong evidence.

#### Lopinavir/ritonavir

Lopinavir/Ritonavir, a kind of viral replication inhibitor, was used for SARS patients [115] and it may be effective for SARS-CoV-2 infection. A recent randomized, controlled, open-label trial [116] included 199 patients with COVID-19 and showed that the time to clinical improvement between lopinavir-ritonavir group and standard-care group was not different (HR = 1.31, 95% CI: 0.95 to 1.80). Besides, 28-day mortality was similar in the two groups, while gastrointestinal adverse events were more common in COVID-19 patients treated with lopinavir/ritonavir at 400/100 mg twice daily. The trial showed disappointing results with lopinavir/ritonavir. However, patients in this study were at the late stage in infection and tissue damage had already appeared, while viral replication inhibitor is more effective in early infection, which may explain inefficacy of the treatment. In addition, Baden et al. [117] also pointed out that the concentration of the drug used in patients failed to inhibit viral replication and both groups were heterogeneous, which may lead to inaccurate conclusion. Therefore, future high-quality blind randomized clinical trials should be carried out to examine the efficacy of lopinavir/ritonavir against COVID-19.

#### Immunomodulatory therapy

There is much attention recently on the use of dexamethasone, tocilizumab and anakinra for COVID-19. In a large RECOVERY trial [118], 2100 COVID-19 patients were enrolled for evaluating the efficacy of dexamethasone for treatment. Surprisingly, it showed that dexamethasone was able to reduce mortality by up to one third in hospitalised patients with severe complication. Dexamethasone, a cheap and widely available steroid, has such a large effect on reducing mortality of COVID-19 patients and it is expected to be an effective and affordable drug for treatment. Tocilizumab, the first IL-6 receptor inhibitor has a significant effect on the treatment of COVID-19 patients. A study [119] demonstrated that tocilizumab improved the clinical outcome in severe and critical patients and it has been recommended to use in severe COVID-19 patients in China. Recently, some randomized controlled trials are being launched, which will provide a more comprehensive knowledge on the use of tocilizumab in COVID-19 patients. A retrospective study [120] with 29 COVID-19 patients found that respiratory function was improved among 72% COVID-19 patients after using high-dose anakinra. Another study [121] which enrolled 8 severe COVID-19 patients with secondary hemophagocytic lymphohistiocytosis also showed the benefits of respiratory function after taking anakinra. However, lager randomized controlled trials are needed to verify the efficacy and safety of anakinra on COVID-19 patients treatment.

### Specific immunotherapy

#### Vaccination

COVID-19 vaccine including nucleic acid vaccine (including mRNA vaccine, DNA vaccine), recombinant genetic engineering (protein recombinant) vaccine, inactivated vaccine, attenuated influenza virus vector vaccine, and adenovirus vector vaccine are yet to be explored [122, 123]. Faced with SARS-CoV-2 infection, global scientific researchers are stepping up the development of vaccines. Coronavirus glycoproteins are potential vaccine targets for SARS-CoV and MERS-CoV. Due to the lack of immunological research on SARS-CoV-2 and its similarity with SARS-CoV, most studies use SARS-CoV immune information to assist the development of a SARS-CoV-2 vaccine. Cytotoxic T-lymphocyte cell epitopes and B cell epitopes on the surface of SARS-CoV-2 are potential targets for the SARS-CoV-2 vaccine [124]. Some researchers think that the entire S protein or the S1 protein containing the RBD is an antigen that can be used for vaccine development [125]; however, some studies have pointed out that vaccines targeting antibodies against S2 linear epitopes may be more effective, because of less genetic mismatches rendering SARS-CoV-derived antibodies ineffective compared with S1 subunit [126].

After the outbreak of SAR-CoV-2, at least 37 biopharmaceutical companies or academic institutions have used multiple platforms including mRNA, DNA, adenoviral vectors, and recombinant proteins to develop preventive vaccines [125]. In China, 5 vaccines (1 for adenovirus vector vaccine, 4 for inactivated vaccines) are under phase II clinical trials. Recently, Zhu et al. [127] published the first inspiring clinical result of vaccine in human. In this dose-escalation, single-center, open-label, non-randomized, phase 1 trial of an Ad5 vectored COVID-19 vaccine, all 108 participants showed immune response after vaccination. From day 14 post-vaccination, rapid specific T-cell responses were found and peak of humoral immunity against SARS-CoV-2 appeared on day 28 post-vaccination. It suggested that the Ad5 vectored COVID-19 vaccine was worth further exploration. Besides, nucleic acid-based vaccines constitute the most advanced strategy in the development of new pathogen vaccines. With the recent improvements in the stability and efficiency of protein translation and the optimization of delivery systems such as lipid nanoparticles (LNPs), nucleic acid vaccines (including DNA and RNA vaccines) are a promising approach that needs further investigation [128, 129]. However, vaccine-mediated harmful immune responses, the time and cost of research and development, the availability of large-scale production, and the ownership and management of vaccines will all be huge challenges that need to be overcome, and strengthening international cooperation is essential for accelerating research to develop new coronavirus vaccines.

#### Passive immunity

Injection of monoclonal antibodies is important for the short-term prevention of viral infections and it is used as an effective treatment upon viral infection. The SARS monoclonal antibody targets the S protein RBD on the SARS-CoV envelope. The RBDs in SARS-CoV-2 and SARS-CoV exhibit homology, prompting speculation that SARS monoclonal antibody is effective against COVID-19. A previous study determined that SARS monoclonal antibody CR3022 bound to the SARS-CoV-2 RBD and the epitope of CR3022 in SARS-CoV-2 RBD did not overlap with the ACE2 binding site. It was believed that CR3022—either alone or in combination with other neutralizing antibodies—might act as a therapeutic candidate for the prevention and treatment of SARS-CoV-2 infection. However, some of the strongest SARS-CoV-specific neutralizing antibodies (such as M396 and CR3014) failed to bind to the SARS-CoV-2 spike protein, establishing that differences in the RBD influenced the cross-reactivity of neutralizing antibodies [130]. It is vital to develop a new monoclonal antibody that can specifically bind to the SARS-CoV-2 RBD.

For patients with rapid disease progression, passive plasma therapy is an effective treatment. WHO recommends the use of convalescent plasma or serum to treat COVID-19 when vaccines or effective antiviral drugs are not available. In China, immune plasma therapy has been clinically effective for patients with severe COVID [131]. However, a randomized clinical trial [132] enrolled 103 patients with severe or life-threatening COVID-19 and showed that compared with the standard treatment group, there was no advancement in time to clinical improvement within 28 days after convalescent plasma therapy. This study was terminated early and was an open-label study, which may be underpowered to explore the differences in result. Further research is expected to provide a more accurate evaluation.

Antibody-dependent enhancement is common in various viruses [133] and it is a focus for vaccine design and passive immunization. Both SARS-CoV and MERS-CoV RBD-specific neutralizing antibodies can mediate antibody-dependent enhancement effects [134]. Whether SARS-CoV-2 exhibits an antibody-dependent enhancement effect remains to be investigated.

### Cell therapy

Cell therapy is expected to emerge as a new way to fight SARS-CoV-2; indeed, projects on stem cell therapy for COVID-19 have been established in China (ChiCTR2000030020). Mesenchymal stem cells have immunomodulatory effects based on their location at the site of inflammation, regulating inflammation-related cytokines and reducing inflammation [135]. Via paracrine cytokines, they are expected to inhibit the cytokine storm and the overwhelming immune response caused by SARS-CoV-2. Consequently, alveolar epithelial cells and vascular endothelial cells are protected. NK cells can also improve human immunity and exert effective antiviral effects. Recently, Food and Drug Administration approved the use of mesenchymal stem cells to treat severe COVID-19 patients and clinical trial of mesenchymal stem cells in the treatment of COVID-19 has also launched in the UK. However, mesenchymal stem cells and NK cells still have a long way to go before their routine use in clinics.

## Conclusion

The outbreak of COVID-19 induced by SARS-CoV-2 has gained much attention worldwide. By June 17, 2020, SARS-CoV-2 infection cases have occurred in as many as 216 countries, areas or territories, and a total of 8,061,550 cases have been confirmed; the scientists are now concentrated on researching the virus for a comprehensive understanding and for the development of preventive and management measures. Here, we summarized the differences between SARS-CoV-2 and SARS-CoV with regards to classification, amino acid composition and protein structure, and epidemiological and pathological characteristics. The pathogenic mechanisms of SARS-CoV-2 have been also discussed. Based on our expertise, we have focused on CVD in patients with COVID-19 and myocardial injury and VTE induced by SARS-CoV-2. Meanwhile, the information of potential medicines and therapies including TCM, chloroquine and hydroxychloroquine, remdesivir, lopinavir/ritonavir and immunomodulatory therapy, and specific immunotherapies and cell therapy have been summarized.

## Data Availability

Not applicable.

## Abbreviations

COVID-19: Coronavirus disease 2019
ORFs: Open reading frames
nsps: Non-structural proteins
RBD: Receptor binding domain
ACE2: Angiotensin converting enzyme2
WHO: World Health Organization
R_0_: Basic reproduction number
ARDS: Acute respiratory distress syndrome
IFN: Interferon
IL: Interleukin
CVD: Cardiovascular disease
VTE: Venous thromboembolism
TCM: Traditional Chinese medicine
